# Semaphorin3A increases M1-like microglia and retinal ganglion cell apoptosis after optic nerve injury

**DOI:** 10.1186/s13578-021-00603-7

**Published:** 2021-05-26

**Authors:** Liu Yun-Jia, Chen Xi, Zhang Jie-Qiong, Zhu Jing-Yi, Lin Sen, Ye Jian

**Affiliations:** Department of Ophthalmology, Daping Hospital, Army Medical Center, Army Medical University, No. 10, Changjiang Branch, Daping, Yuzhong District, Chongqing, 400042 People’s Republic of China

**Keywords:** semaphorin3A (Sema3A), retinal ganglion cells (RGCs), Microglia polarization, M1/M2-like, Neuroinflammation

## Abstract

**Background:**

The mechanisms leading to retinal ganglion cell (RGC) death after optic nerve injury have not been fully elucidated. Current evidence indicates that microglial activation and M1- and M2-like dynamics may be an important factor in RGC apoptosis after optic nerve crush (ONC). Semaphorin3A (Sema3A) is a classic axonal guidance protein,which has been found to have a role in neuroinflammation processes. In this study, we investigated the contribution of microglial-derived Sema3A to progressive RGC apoptosis through regulating paradigm of M1- and M2-like microglia after ONC.

**Method:**

A mouse ONC model and a primary microglial-RGC co-culture system were used in the present study. The expression of M1- and M2-like microglial activation markers were assessed by real-time polymerase chain reaction (RT-qPCR). Histological and Western blot (WB) analyses were used to investigate the polarization patterns of microglia transitions and the levels of Sema3A. RGC apoptosis was investigated by TUNEL staining and caspase-3 detection.

**Results:**

Levels of Sema3A in the mouse retina increased after ONC. Treatment of mice with the stimulating factor 1 receptor antagonist PLX3397 resulted in a decrease of retinal microglia. The levels of CD16/32 (M1) were up-regulated at days 3 and 7 post-ONC. However, CD206 (M2) declined on day 7 after ONC. Exposure to anti-Sema3A antibodies (anti-Sema3A) resulted in a decrease in the number of M1-like microglia, an increase in the number of M2-like microglia, and the amelioration of RGC apoptosis.

**Conclusions:**

An increase in microglia-derived Sema3A in the retina after ONC partially leads to a continuous increase of M1-like microglia and plays an important role in RGC apoptosis. Inhibition of Sema3A activity may be a novel approach to the prevention of RGC apoptosis after optic nerve injury.

**Supplementary Information:**

The online version contains supplementary material available at 10.1186/s13578-021-00603-7.

## Introduction

Optic nerve injury resulting in progressive retinal ganglion cell (RGC) death is a serious and irreversible cause of blindness [1]. Retinal neuroinflammation is a leading factor limiting the recovery of RGC after primary optic nerve impairment [2, 3]. Microglia makes up a significant portion of the resident glial population in the retina and are key mediators of neuroinflammation [2, 4, 5]. In previous studies, we have investigated the essential role of microglia in triggering retinal inflammation [6, 7]. Optic nerve injury is followed by migration, activation, and proliferation of microglia [8, 9]. Activated microglia, including retinal microglia, can be divided into two major types: pro-inflammatory type M1-like microglia and anti-inflammatory type M2-like microglia [10, 11]. M1-like microglia secrete pro-inflammatory cytokines, including TNFα, IL-23, IL-1, and IL-12, which contribute to neuronal damage. M2-like microglia, activated by IL-4 or IL-13, are anti-inflammatory and promote tissue repair and wound healing [4]. Sufficient evidence has suggested that reciprocal transformation of M1-like and M2-like microglia occurs under certain conditions. This reciprocal transformation can lead to either the increase or the subsidence of neuronal inflammation [12–14]. However, the initiating factors governing the polarization of microglia in RGC secondary injury is still not fully elucidated.

Sema3A is involved in the negative regulation of neuronal axon and dendrite polarity [15–19] as well as acting as an effective regulator in some essential stages of inflammation and the immune response [16, 20–22]. For example, patients suffering from late-stage proliferative diabetic retinopathy have elevated levels of vitreous Sema3A, which attracts neuropilin-1(NRP-1) positive mononuclear phagocytes [23]. Sema3A also activates the transcription factor NF-κB via the TLR4 signaling pathway in macrophages to increase pro-inflammatory cytokine production and to augment inflammatory responses in a sepsis-induced cytokine storm [24]. Previous studies have confirmed that the level of Sema3A (mainly distributed in the RGC layer of the retina) increases significantly during the 3 days after optical nerve crush (ONC). Its expression can persist for 14–28 days after injury [19, 25]. Anti-Sema3A antibodies (anti-Sema3A) can rescue RGCs from apotosis that occurs after optic nerve axotomy [26]. Neuropilin 1 (Nrp1) is a receptor of Sema3A. It has been shown that levels of microglia in Nrp1-floxed mouse retina are significantly lower than in the retinas of WT mice [23]. Nrp1^+^ microglia are present throughout the retina during vascular development, although they are more prevalent in non-vascularized retinal tissue [27]. This suggests a potential paracrine effect of microglial Sema3A/Nrp1 signaling in retinal development and pathogenesis. However, the role of Sema3A in retinal neuroinflammation and its interaction with retinal microglia remain unclear.

In current study, we investigate the regulatory effect of Sema3A on M1- and M2-like microglia dynamics in vivo and in vitro. In a mouse ONC model, we demonstrate that a significant amount of Sema3A is secreted by retinal microglia post-ONC. The dynamic changes of M1/M2-like microglia and neuronal apoptosis were evaluated. The levels of M1/M2-like microglia and the extent of RGC apoptosis were further verified in a co-culture model of primary microglia and RGCs. We found that the increases of Sema3A expression increased the pro-inflammatory M1-like phenotype and decreased the anti-inflammatory M2-like phenotype. In consequence, as pro-inflammatory cytokine release increased, RGC underwent apoptosis. anti-Sema3A treatment in vitro and in vivo decreased the M1-like phenotype and increased the M2-like phenotype, contributed to the amelioration of RGC apoptosis.

## Materials and methods

### Animals and surgery

All animals were treated according to the ARVO Statement for Use of Animals in Ophthalmic and Vision Research. Experimental procedures were approved by the Institutional Animal Care and Use Committee of the Army Medical University.

**Fig. 1:**
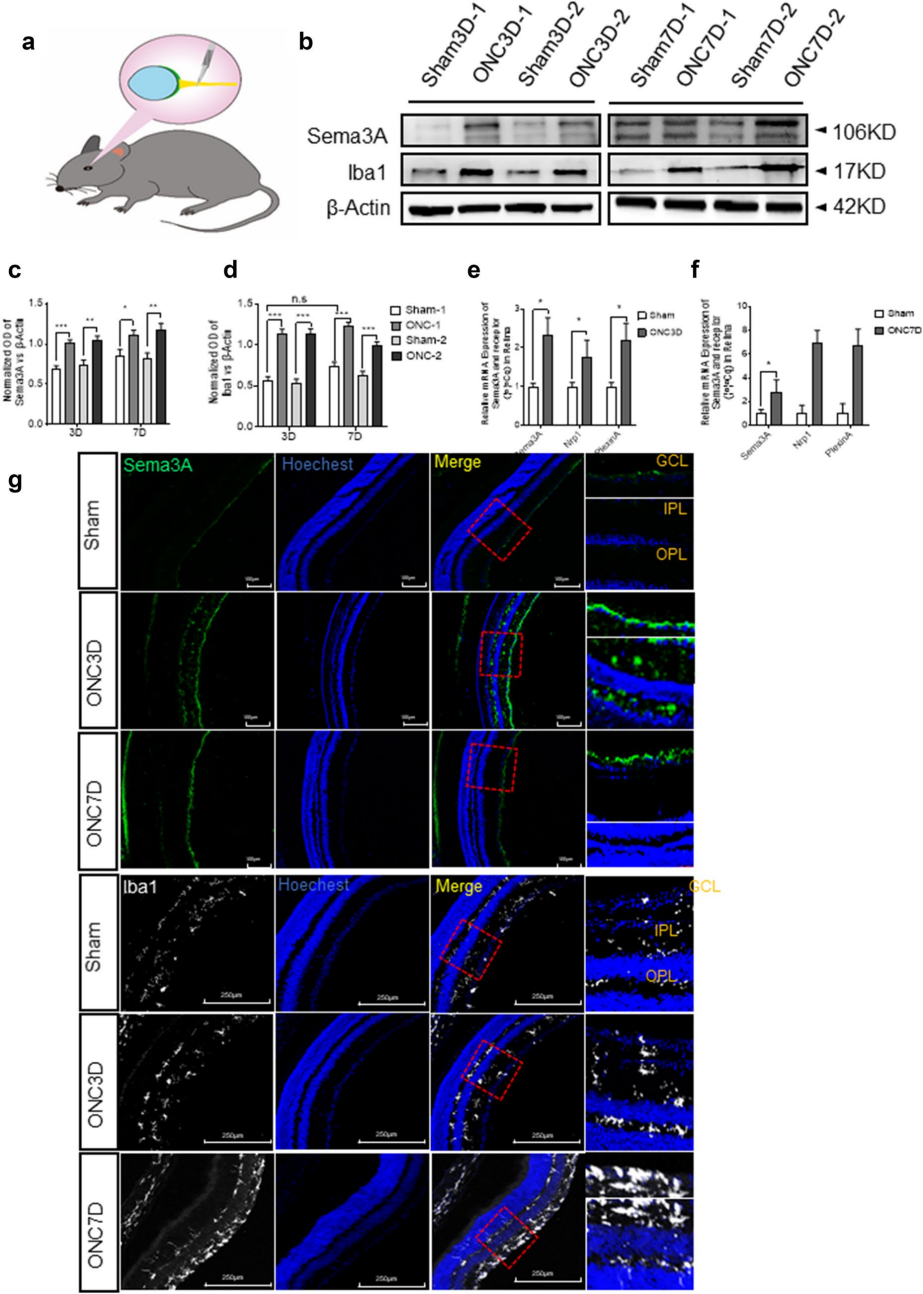
Sema3A is increased in ONC mice retina and is accompanied by microglia activation. **a** Schematic diagram of the ONC model demonstrating the site of ONC. **b** Western blot of Sema3A and Iba1 expression in retina at 3 and 7 days (3D and 7D) after ONC. β-actin was used as a loading control. **c**, **d** Quantitative western blot analysis indicates a significant up-regulation of Sema3A and Iba1 (Mean ± SEM, n = 10). **e**, **f** mRNA expression of Sema3A and its receptors in retina at 3 and 7 days post-ONC as measured by quantitative RT-qPCR. Gene expressions were normalized to β-actin. The results are representative of ten independent experiments and are shown as mean ± SEM (n = 10) (G) Representative confocal images showing immunostaining of Sema3A and Iba1 in retinal cryosections. Sema3A was mainly detected in the ganglion cell layer (GCL) and Iba1 was mainly distributed in GCL, IPL and OPL. Scale bar = 250 μm

C57BL/6 mice were purchased from the Army Medical University. The mice were housed at an animal care facility with a 12/12-h light/dark cycle and *ad libitum* access to food and water. The classic model of optic nerve crush (ONC) was performed as previously described [6, 28]. Adult C57BL/6J mice (male, aged 6–8 weeks; weight: 20–24 g) were anesthetized by intraperitoneal injection of pentobarbital sodium (30 mg/kg). The exposed optic nerve of the left eye was crushed for 10 s at a distance of 1.5 mm from the eye globe with ultrafine self-closing forceps without damage to the retinal vessels or the blood supply. The right eye was used as a sham control (Fig. 1a). The mice were killed at 3 and 7 days post-ONC. Primary microglia were cultured from newborn C57BL/6 mouse cortex for the in vitro studies.

### Microglia depletion

The colony-stimulating factor 1 receptor antagonist PLX3397 was used for the pharmaceutical depletion of microglia. Male mice aged 8–10 weeks were given AIN-76 A chow containing 290 mg/kg PLX-3397 [29, 30]. Age-matched controls were given AIN-76 A chow without PLX-3397. After 3 days of diet administration, the mice underwent the ONC procedure and were sacrificed.

### Intravitreous injections

Adult mice received intravitreous injections of anti-Sema3A (1 µl, neutralizing antibody) [31] in their left eyes before the ONC procedure. The right eyes were used as sham controls and received saline injections. The intravitreal injection procedure was performed as described previously [32] without elevated intraocular pressure detected in any of the eyes after surgery.

### Immunostaining

Primary antibodies included anti-Sema3A (1:100, rabbit monoclonal, Abcam, ab23393), anti-CD16/32 (1:100, goat polyclonal, R&D, AF1460), anti-CD206 (1:100, mouse monoclonal, Abcam, ab8918), anti-Iba1 (1:100, rabbit monoclonal, Abcam, ab178846), and anti-P2RY12 (5 µg/ml, mouse monoclonal, Biolegend, 848001). Secondary antibodies included anti-mouse IgG, anti-goat IgG, anti-Rat IgG, and Alexa Fluor 488, 594, and 647 conjugated to anti-rabbit IgGs (1:1000, Invitrogen). Mouse retinas were fixed in 4 % paraformaldehyde and cryo-sectioned at a thickness of 10 μm. Retina cryosections and fixed primary microglia were incubated with 0.1 % Triton X-100 (Sigma-Aldrich) in PBS at room temperature for 10 min, then incubated with primary antibodies at 4 °C overnight. Secondary antibodies were applied for 1 h at room temperature. The slides were mounted after nuclei counterstaining (Hoechst 1:2000). Images were taken by an SP-8 confocal microscope (Leica, Germany). For quantification of immunofluorescence, CD16/32^+^, CD206^+^, and Iba1^+^ cells were counted in 3 s. (10 μm thick) of retinal tissue per mouse. The mean number of cells per field was determined. All data were expressed as mean (n = 3 mice per group) ± SEM. The sections were selected randomly and were analyzed under a Leica SP8 confocal laser scanning microscope. Quantification of cell numbers was analyzed using ImageJ software.

### Whole-mounted retinal immunofluorescence

Retinas were fixed in 4% PFA for 1 h, then dissected as whole-mounts. An orientation record was maintained as previously described [28]. Briefly, the intact retinas were incubated in PBS containing 5% BSA and 3% Triton-X-100 at 4 °C overnight. Primary mouse monoclonal anti-Tuj1 antibody (Covance, Cat. MMS435P) was added (1:500) and incubated overnight at 4 °C. The retinas were incubated with secondary anti-mouse IgG antibodies conjugated to Alexa Fluor 488 overnight at 4 °C and examined by confocal microscopy with the appropriate filters (SP8, Leica, Germany).

### Microglial cell culture

Mixed glial cultures were isolated from the cerebral cortices of 1-day-old C57BL/6 mice as previously described [33]. Cells were dissociated under aseptic conditions, suspended in DMEM-F12 with 10 % FBS, and seeded at a density of 62,500 cells/cm^2^ [34]. Cells were cultured at 37 °C and 5 % CO_2_ for 15 days. Mixed glial cells were then shaken at 200 rpm in a rotary incubator overnight at 37 °C to dissociate the cells. The suspended cells were collected and replanted in DMEM-F12 with 10 % FBS. The purity of the microglia was confirmed by immunostaining using anti-Iba1. Then, three independent microglial cultures were treated for 6 h with vehicle (cell culture medium) Sema3A (R&D) and 100 ng/mL of LPS (026:B6 *Escherichia coli* serotype, Sigma Aldrich), and primary microglia were harvested after 1 day and 3 days for further experiments.

### Immunoblotting

Western blot (WB) analysis was performed on protein extracted from post-ONC mouse retinas, primary microglia, and RGCs as previously described [28]. Washed and blocked polyvinylidene fluoride membranes from WBs were incubated with primary antibodies. Primary antibodies included anti-Sema3A (1:1000, rabbit monoclonal, Abcam, ab23393), anti-CD16/32 (1:1000, goat polyclonal, R&D, AF1460), anti-CD206 (1:500, rabbit monoclonal, Abcam, ab125028), anti-Iba1 (1:1000, rabbit monoclonal, Abcam, ab178846), anti-SMI32(1:1000, rabbit monoclonal, Abcam, ab207176), and anti-Map2 (1:1000, rabbit monoclonal, CST, #4542). Membranes were then incubated with horseradish peroxidase-conjugated anti-rabbit, anti-mouse, or anti-goat IgG secondary antibodies (Santa Cruz Biotechnology Inc., Santa Cruz, CA, United States) in TBS-T for 1.5 h at room temperature. ECL-Plus reagent (Bioground) was applied to the membranes and chemiluminescence visualized by a Fluor-S-max imager (Bio-Rad).

### RT-qPCR

Total RNA was extracted from retinas and primary microglia using the RNeasy RNA isolation kit (Qiagen). Reverse transcription reactions were performed using the PrimeScript™ RT reagent Kit (Takara) according to the company’s instructions. RT-qPCR was performed using the SYBR method (Quanta BioSciences) on a CFX96 Real-Time PCR Detection/C1000 Thermal Cycler system (Bio-Rad). The results were normalized to β-actin. Relative quantification was calculated using the 2−ΔΔCq method. The primer sequences used are listed in Table 1.

**Table 1:** Primer sequences used for RT-qPCR

Gene	Forward (5’ -3’)	Reverse (5’ -3’ )
Sema3A	TGGGATTGCCTGTCTTTT	GGCCAAGCCATTAAAAGTGA
Nrp1	GACAAATGTGGCGGGACCATA	TGGATTAGCCATTCACACTTCTC
PlexinA	GAATGCAAATGGGCTGGAAAAG	GATAGCGAAGTCCCGTCCCA
CD86	ACTTACGGAAGCACCCACG	CTTTGTAAATGGGCACGGC
IL-1-β	ACGGACCCCAAAAGATGAAG	AGGCCACAGGTATTTTGTCG
IL-6	ATACCACTCCCAACAGACC	TGCATCATCGTTGTTCATAC
iNOS	GCCACGGACGAGACGGATAG	GGGCACATGCAAGGAAGGG
TNF-α	CCTCTTCTCATTCCTGCTTG	TCTGGGCCATAGAACTGATG
CD206	CCACGGATGACCTGTGCTC	GGTTCCACACCAGAGCCATC
Arg1	CAACACTCCCCTGACAACC	ACGATGTCTTTGGCAGATATG
IL-10	AGCCGGGAAGACAATAACTG	GGAGTCGGTTAGCAGTATGTTG
Ym1	TGCGTGACTATGAAGCATTG	TGACGGTTCTGAGGAGTAGAG
Actin	TTCTACAATGAGCTGCGTGTG	CCATCACAATGCCTGTGGT

### Statistical analysis

Statistical analyses were performed using GraphPad Prism 7.0 software (GraphPad, La Jolla, CA, U.S.A.) and reported as mean ± S.E.M. Student’s t-test, one-way ANOVA, and least significant difference post-hoc tests were also employed. All experiments were performed independently with n > 3. P values ≤ 0.05 were considered to be statistically significant. Significant values were marked as * (P values below 0.05), ** (P values below 0.01), and *** (P values below 0.001).

## Results

### After optic nerve crush, Sema3A increased, accompanied by microglial activation

WB results showed that Sema3A expression in the mouse retina increased significantly and peaked at 3 days post-ONC. On day 7 post-ONC, Sema3A levels declined but remained higher than the control (Fig. 1b), consistent with previous results [19]. At 3- and 7-days post-injury, there was a concomitant increase in expression of Iba1, indicating that microglia were significantly activated (Fig. 1d). Based on the RT-qPCR analysis, there was a significant increase in mRNA expression of Sema3A and its receptor Nrp1 at 3 days and 7 days post-ONC (Fig. 1e, f). Sema3A immunofluorescence staining was primarily located in the Ganglion Cell layer (GCL) (Fig. 1g) while Iba1 was distributed throughout the GCL, inner plexiform (IPL), and outer plexiform (OPL) layers. Detailed morphological analysis at 3- and 7-days post-ONC indicated that Iba1^+^ cells adopted a more irregular soma shape, with increased cell body size and retracted processes.

### Microglia depletion reduces Sema3A levels after ONC

**Fig. 2:**
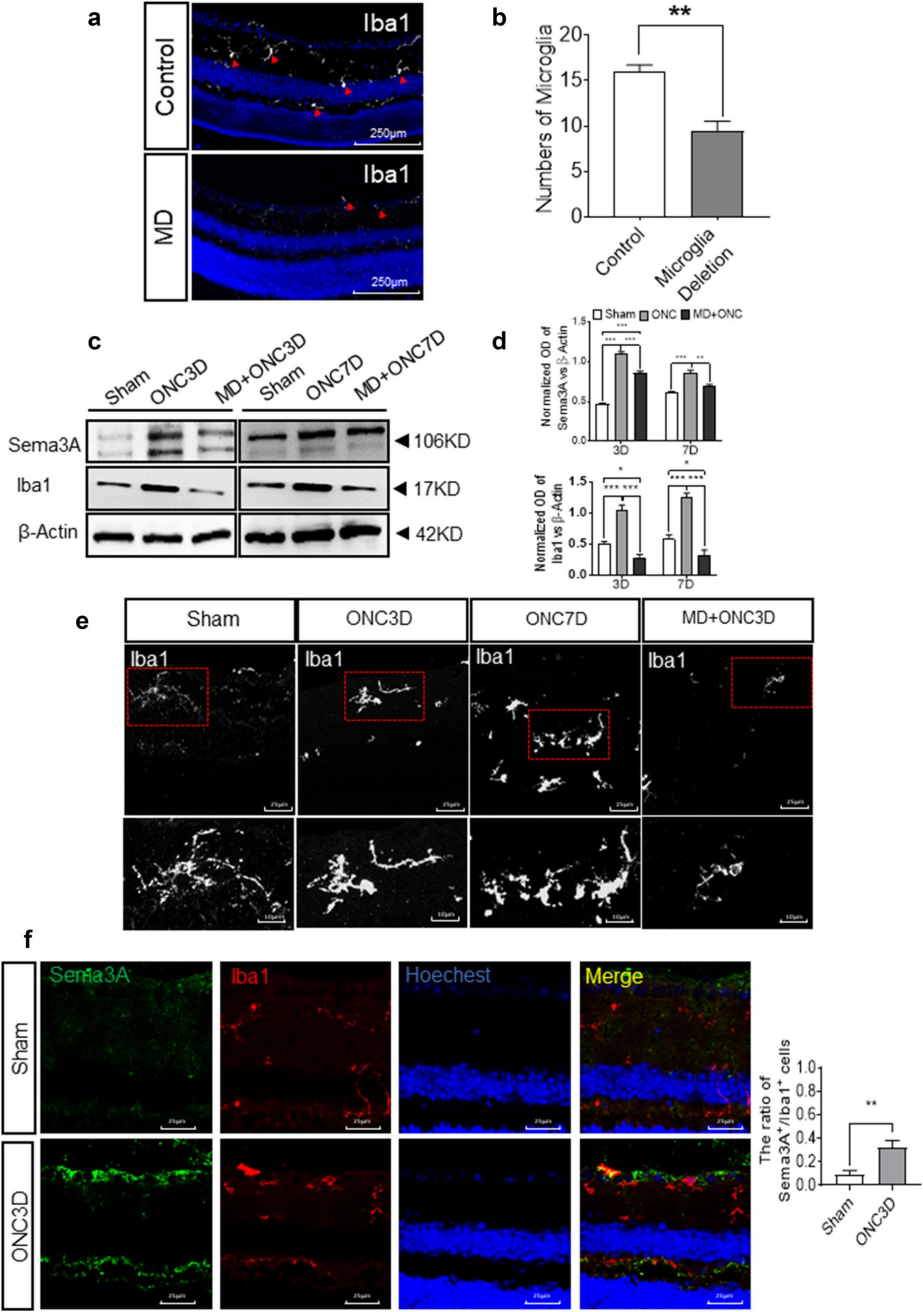
Sema3A is significantly reduced in microglia depleted mice retina. **a** Representative confocal images showing immunostaining of Iba1 (red arrows) with Hoechst nuclear staining. Scale bar = 250 μm. **b** Quantification of positive Iba1 in sham and microglia depletion groups (n = 4). Iba1 staining was reduced after PLX-3397 treatment. **c**, **d** Western Blot shows the expression levels of Sema3A and Iba1 in retina after microglia depletion (MD). β-actin was used as a loading control. Quantitative analysis indicates a significant down-regulation of Sema3A in MD group (Mean ± SEM, n = 5). **e** Representative confocal images showing immunostaining of Iba1. Scale bar = 25 μm. **f** Representative confocal images showing immunostaining of Sema3A (green) and Iba1(red) in retinal cryosections. Scale bar = 25 μm

Sema3A can be produced by stressed neurons, activated astrocytes, and microglia [35–37]. To determine the contribution of Sema3A from microglia in the mouse model, mice treated with PLX3397, which depleted microglia in retina [30, 38, 39], underwent ONC. Immunofluorescence staining revealed a significant 50 % reduction in retinal microglia after PLX3397 treatment (Fig. 2a, b). WB results showed that Sema3A and Iba1 levels in retinal microglia from PLX-3397 treated mice were both significantly lower than that in untreated mice (Fig. 2c, d), suggesting that microglia serve as one of the sources of Sema3A protein. However, the remaining microglia were activated, similar to microglia in untreated ONC mice (Fig. 2e). Immunostaining of Sema3A and Iba1 in retinal cells showed that Sema3A protein co-localized with Iba1 in microglial cells (Fig. 2f).

### M1-like microglia levels increase and M2-like microglia levels decrease in retinal tissues post-ONC, similar to cultured primary microglia treated with Sema3A

**Fig. 3:**
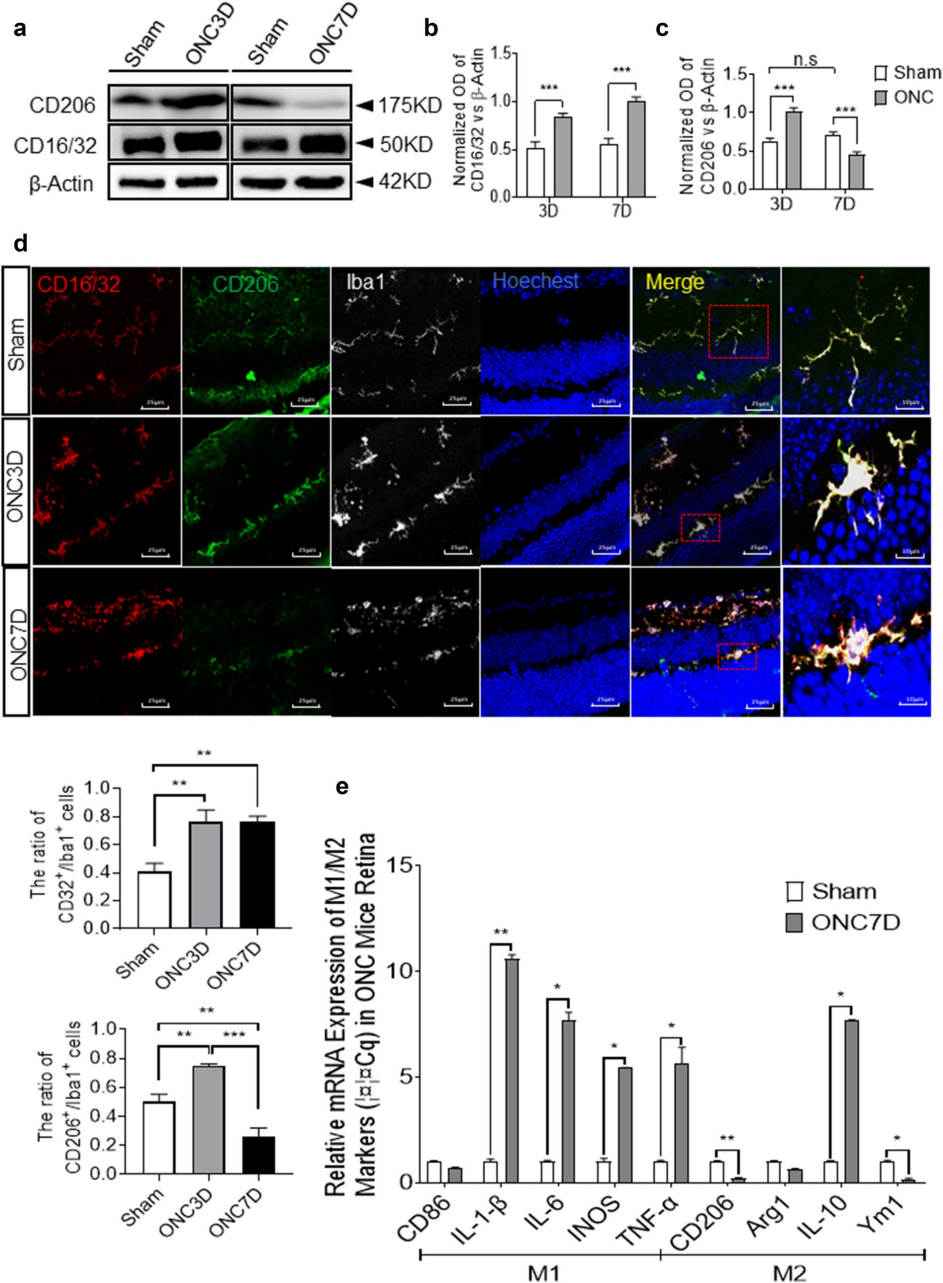
At 7 days post-ONC, activated microglia show significantly increased M1-like phenotype and decreased in M2-like phenotype. **a **Expression of CD16/32 and CD206 in the retina at 3 and 7 days post-ONC. **b**, **c** Quantitative analysis indicates a significant increase of CD16/32and CD206 at 3 days post-injury. Level of CD16/32 continuous to increase at 7 days post-ONC, while CD206 decreased significantly. (Mean ± SEM, n = 12). **d** Representative confocal images showing immunofluorescent staining of CD16/32 (red), CD206 (green) and Iba1 (white) with Hoechst (blue) nuclear staining in retina cells of sham, ONC3D, and ONC7D groups. Scale bar = 25 μm. Quantitative analysis indicates a significant increase of CD206 at 3 days post-injury, and an increase of CD32 both at 3 days and 7days post-injury. Ratio of the number of double positive CD32/Iba1 cells vs. Iba1 positive cells (bar graph). Ratio of the number of double positive CD206/Iba1 cells vs. Iba1 positive cells (bar graph). **e** mRNA expression of cytokines secreted by microglia in retina at 7 days post-ONC measured by RT-qPCR. Gene expressions were normalized to β-actin. The results are representative of three independent experiments and are shown as mean ± SEM (n = 3)

**Fig. 4:**
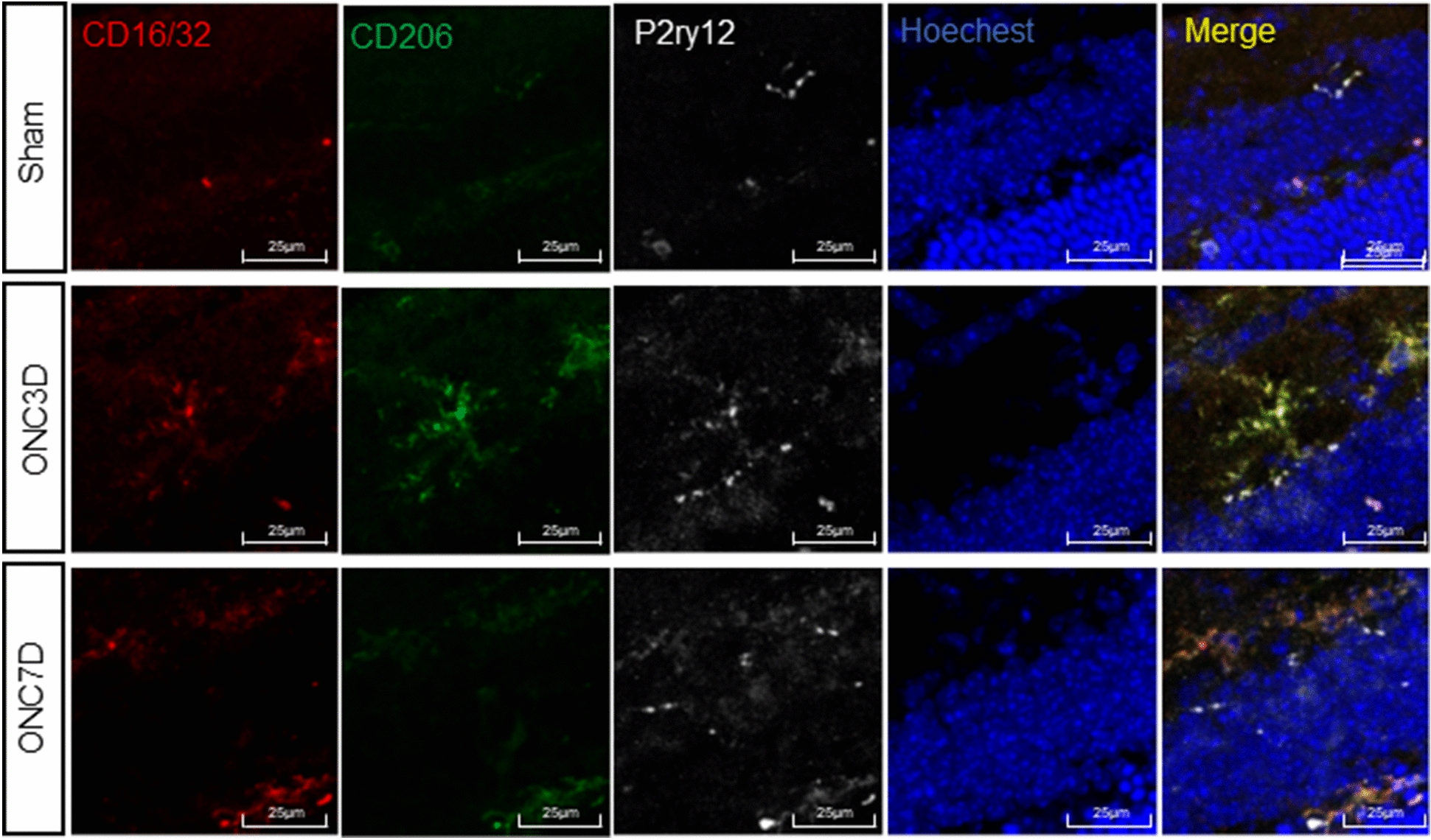
Microglia polarization after optic nerve injury mainly occurred in resident microglia. Representative confocal images and quantitative analysis showing immunofluorescent staining of CD206 (green), P2ry12(white) CD16/32 (red) with Hoechst (blue) nuclear staining in retina cells at 3 days and 7 days post-ONC, Scale bar = 25 μm

There was robust induction of the M1-like marker CD16/32 and the M2-like marker CD206 in the retina at 3 days post-ONC (Fig. 3a–c). At 7 days post-ONC, the level of CD16/32 (M1-like) was observed to be higher than that observed at 3 days post-ONC. However, the expression levels of CD206 were greatly reduced (Fig. 3a–c), which was confirmed by immunofluorescence staining (Fig. 3d). RT-qPCR revealed that cytokines secreted by M1-like microglia, including IL1-β, IL-6, iNOS, and TNF-α, were significantly up-regulated, while those of M2-like microglia, such as CD206 and Ym1, were down-regulated (Fig. 3e). Cell staining with P2ry12 co-labeled with CD16/32 and CD206 was used to distinguish resident microglia from infiltrating macrophages. The results suggested that microglia polarization after optic nerve injury mainly occurred in resident microglia (Fig. 4).

**Fig. 5:**
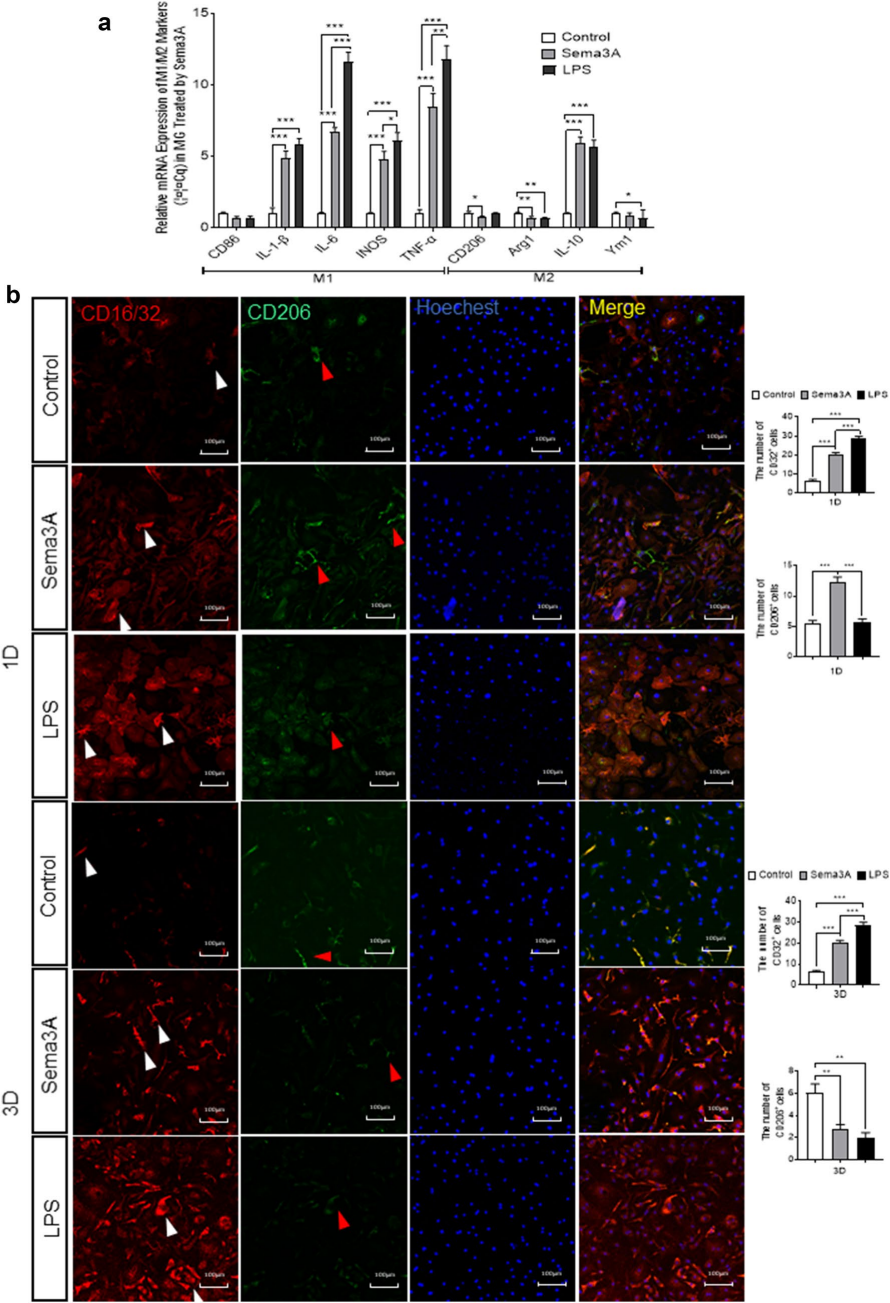
Increased M1-like and decreased M2-like phenotypes observed in primary microglia treated with Sema3A. **a** mRNA expression of cytokines secreted by primary microglia treated with Sema3A. Gene expressions were normalized to β-actin. The results are representative of three independent experiments and are shown as mean ± SEM (n = 3). **b**, **c** Representative confocal images and quantitative analysis showing immunofluorescent staining of CD16/32 (red, white arrow) and CD206 (green, white arrow) with Hoechst (blue) nuclear staining in primary microglia 1 day and 3 days after Sema3A and LPS treatment. Scale bar = 100 μm

In vitro studies demonstrated that the treatment of primary microglial cells with Sema3A significantly increased the population of M1-like microglia (Fig. 5a). Double-immunofluorescence staining of CD16/32 and CD206 in primary microglia showed that 1 day after Sema3A and LPS treatment, CD16/32 + and CD206 + cells were both increased. However, 3 days after Sema3A and LPS treatments, an extra immunofluorescent staining in primary microglia showed increased M1-like microglia and decreased M2-like microglia, which supported the RT-qPCR results (Fig. 5b, c). These in vitro results indicate that Sema3A is involved in the pathological processes that increase the population of M1-like microglia and decrease the population of M2-like microglia after ONC.

### Anti-Sema3A administration significantly reduces RGCs apoptosis in vivo and in vitro

**Fig. 6:**
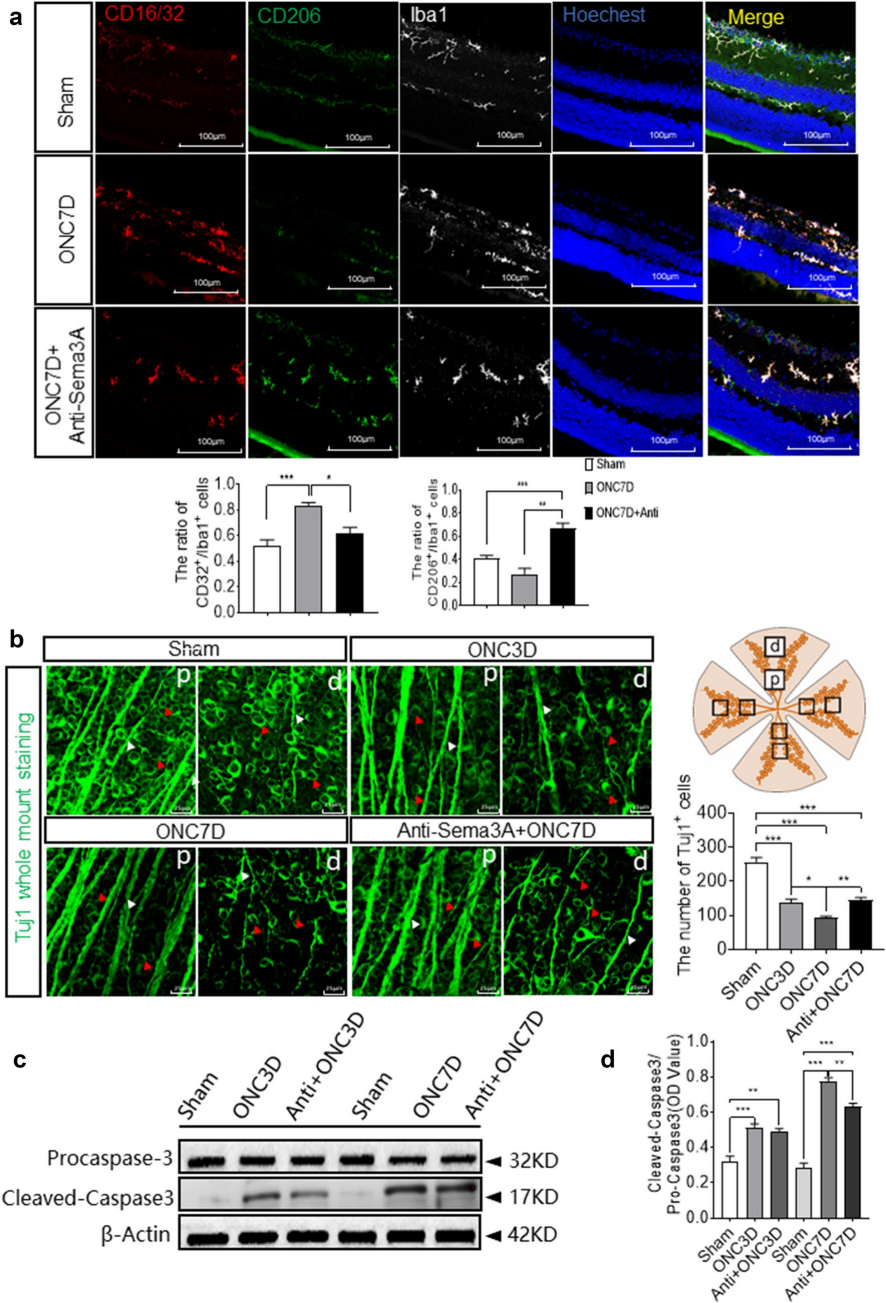
Intravitreal injection of anti-Sema3A increased M2-like microglia and decreased RGCs apoptosis in the retina. **a** Representative confocal images and quantitative analysis showing immunofluorescent staining of CD206 (green), CD16/32 (red) and Iba1 (white) with Hoechst (blue) nuclear staining in retina from mice post-injury at 7 days with or without anti-Sema3A treatment. Scale bar = 100 μm. Quantitative analysis indicates a significant increase of CD206 with anti-Sema3A treatment and CD32 without anti-Sema3A at 7 days post-injury (Mean ± SEM, n = 5). Ratio of the number of double positive CD32/Iba1 cells vs. Iba1 positive cells (bar graph). Ratio of the number of double positive CD206/Iba1 cells vs. Iba1 positive cells (bar graph). **b** Representative confocal images of anti-Tuj1 and the quantitative analysis (n = 4) show reduction in RGCs (red arrows) and axons (white arrows) after ONC. anti-Sema3A alleviated RGCs apoptosis. Scale bar = 25 μm. **c**, **d** Expression of Pro-caspase3 and cleaved-caspase3 in the retina. Quantitative analysis indicates a significant increase of cleaved-caspase3 in the ONC group, which is reduced after anti-Sema3A injection (n = 6). β-actin was used as a loading control

Sema3A has been shown to rescue RGCs from cell death following optic nerve axotomy [26], yet the mechanisms vary. We performed intravitreal injections of anti-Sema3A and observed that, on day 7 post-ONC, the increase in the number of M1-like polarized cells slowed but the number of M2-like cells continued to increase (Fig. 6a). In the absence of anti-Sema3A treatment, whole-mounted retinal immunofluorescence staining with anti-Tuj1 antibody indicated that the number of RGCs in the retina had decreased significantly at days 3 and 7 post-ONC. Although no significant difference in RGC numbers was detected between the ONC3D group (3-day post-ONC) and the anti-Sema3A + ONC3D group, the numbers of RGCs in the ONC7D group were significantly lower than that of the anti-Sema3A + ONC7D group (Fig. 6b). These results indicate that intravitreal treatment with anti-Sema3A reduces M1-like microglia polarization, increases M2-like level, and reduces the loss of RGCs following ONC.

We measured cleaved caspase-3 levels to investigate the anti-apoptotic effect of anti-Sema3A treatment on RGCs on day 7 post-ONC [28, 40]. Cleaved caspase-3 protein levels were significantly reduced in the anti-Sema3A + ONC7D group, indicating that anti-Sema3A protected RGCs from cleaved caspase-3-induced apoptosis (Fig. 6c, d). TUNEL staining and Bax/BCL-2 blotting also suggest the protection of RGCs by anti-Sema3A (Additional file 1: Fig. S1A, B).

**Fig. 7:**
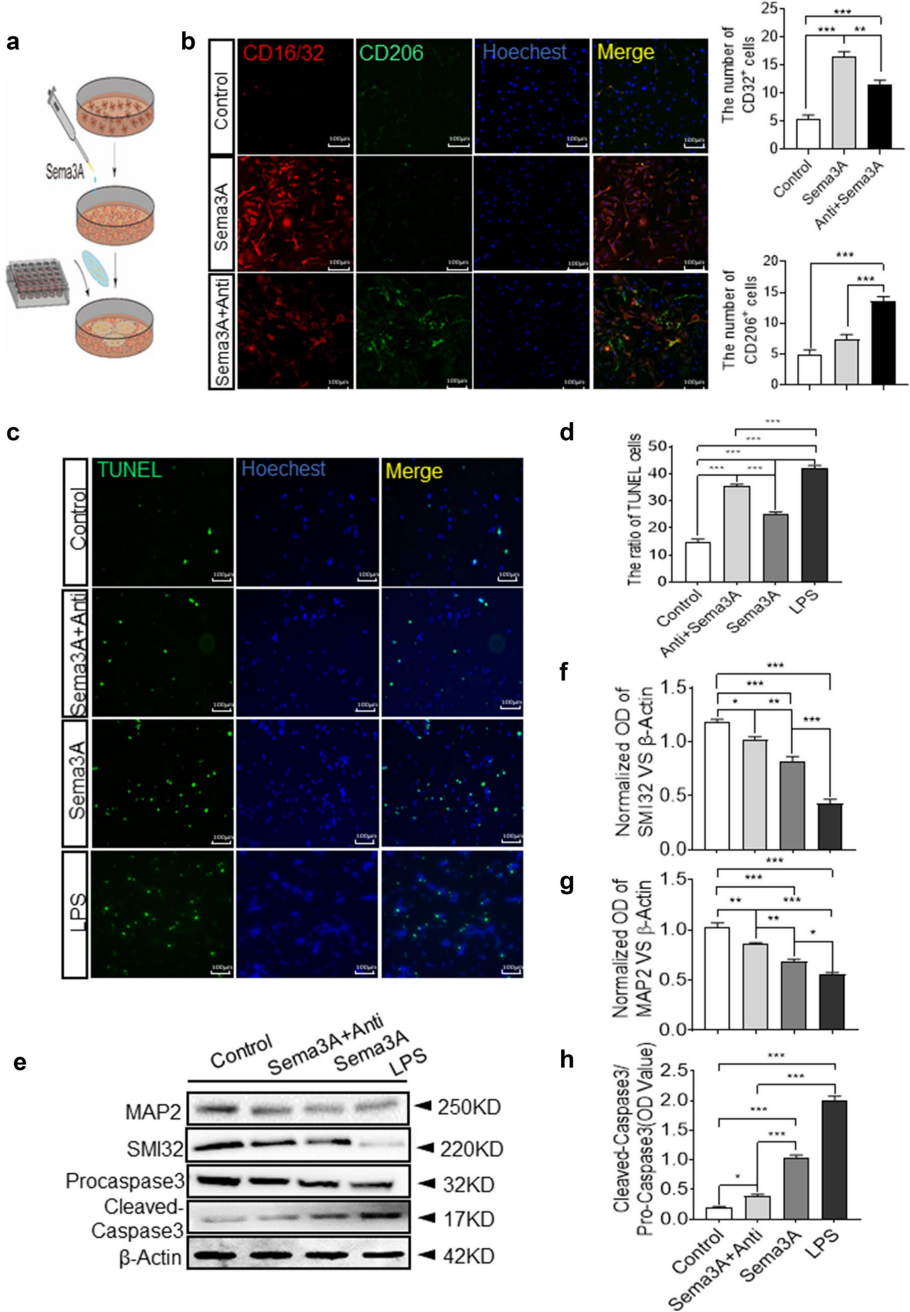
Anti-Sema3A treatment in primary microglia increased M2-like microglia and decreased RGCs apoptosis in primary microglia and RGC co-culture. **a** Schematic diagram of primary microglia and RGC co-culture system. **b** Representative confocal images showing immunofluorescent staining of CD206 (green) and CD16/32 (red) with Hoechst (blue) nuclear staining in Sema3A treated primary microglia with or without anti-Sema3A treatment. Scale bar = 100 μm. Quantitative analysis indicates a significant increase of CD206 with anti-Sema3A treatment. (Mean ± SEM, n = 6). **c** TUNEL staining of RGCs in control, Sema3A + anti-Sema3A, Sema3A, and LPS groups. Scale bar = 100 μm. **d** Quantification of TUNEL-positive cells vs. Hoechst staining (n = 4). **e–h** Western Blot and quantitative analysis show expression of SMI32, MAP2, pro-caspase3, and cleaved-caspase3 in RGCs co-cultured with microglia. β-actin was used as a loading control. (Mean ± SEM, n = 5)

Both Sema3A- and LPS-treated microglia induced RGCs apoptosis in an in vitro primary microglial-RGC co-culture model (Additional file 1: Fig. S1A). Anti-Sema3A + Sema3A-treated co-cultures exhibited decreased M1-like microglia levels, increased M2-like microglia levels, and decreased RGCs apoptosis (Additional file 1^1^ Fig. S1B). In the Sema3A- and LPS-treated co-cultures, SMI32 and MAP2 proteins were significantly lower than controls. In the anti-Sema3A treated co-cultures, levels of cleaved caspase-3 were significantly reduced compared to Sema3A- and LPS-treated groups (Fig. 7d, e).

## Discussion

Visual impairment caused by optic neuropathy is a primary cause of blindness. The mechanisms of irreversible RGCs death from conditions such as optic nerve injury and glaucoma remain unclear. Previous investigations have suggested that Sema3A plays a crucial role in the development of retinal inflammation and RGCs apoptosis post-ONC [24, 26].

Sema3A is continuously expressed in the retina from development through adulthood [41, 42]. It is known that Sema3A is significantly up-regulated in the retina post-ONC [19]. Our results demonstrated that the peak expression of retinal Sema3A occurs at day 3 post-ONC and continues to be expressed for 14–28 days post-injury [43] (Fig. 1b–f). Astrocytes, endothelial cells, and activated microglia contribute to the increase of Sema3A secretion [44, 45] (Fig. 2). This is confirmed by the observation that as the number of retinal microglia declined with PLX3397 treatment, a significant decrease of Sema3A was observed in the retina.

Sema3A causes the collapse of the growth cone of regenerated axons and inhibits axon elongation by binding to the receptor complex Nrp1/PlexinA [19]. Blocking Sema3A binding to Nrp1/PlexinA effectively reduces growth cone collapse of the dorsal root ganglion after spinal cord injury and promotes nerve regeneration in a rat olfactory nerve axotomy model [46]. Emerging evidence suggests that Sema3A regulates B and T lymphocytes and contributes to the progression and development of diseases such as Systemic Lupus Erythematosus and cancer [47, 48]. Sema3A also plays a vital role in the migration and transportation of dendritic cells and the recruitment of mononuclear phagocytes [49]. Our results show that microglia are activated and the number of pro-inflammatory M1-like cells are increased with increasing retinal Sema3A. At the same time, the number of anti-inflammatory M2-like microglia is decreased, accommodating the microglial polarization M1-/M2-like dynamic response and primarily occurring in resident microglia (Figs. 3, 4 and 5). This finding extends the current understanding of the interaction between Sema3A and microglia and demonstrates the important role of Sema3A in neuroinflammation following optic nerve injury.

Anti-Sema3A inhibition of Sema3A significantly reduced the rate of M1-like cell polarization and increased the rate of M2-like microglia polarization post-ONC in the retina (Fig. 6a). Similar results were obtained in vitro using primary microglia cultured with Sema3A protein (Fig. 7). RGCs apoptosis in mouse retina post-ONC and in primary microglial-RGCs co-culture was reduced by anti-Sema3A treatment (Figs. 5, 6 and 7, Additional file 1: Fig. S1). These findings demonstrate that elevated Sema3A induces RGCs apoptosis and inhibits the regeneration of RGCs axons by regulating M1/M2-like microglia dynamics post-ONC (Figs. 1, 3, 4, 5, 6 and 7). Our results shed light on part of the RGCs apoptosis pathway induced by Sema3A post-ONC and suggest that Sema3A regulation is a possible new approach for the treatment of optic nerve injury.

Microglia can be polarized along a continuum toward an inflammatory (M1) or a non-inflammatory (M2) state and microglial reciprocal transformation may participate in the progression of neurodegenerative diseases, such as Alzheimer’s disease [14, 50]. After spinal cord injury, TNF prevents the phagocytosis-mediated conversion of M1-like to M2-like cells and mediates an increase in iron-induced changes of IL-4-polarized M2 cells to M1 cells, which is detrimental to recovery [13, 51]. Our results show that an increase in Sema3A levels post-ONC leads to a significant up-regulation of M1-like microglia and a concomitant decrease in M2-like microglia, consistent with the dynamic changes of M1/M2 phenotype after traumatic brain injury and stroke. The regulation of M1/M2-like microglia dynamics by Sema3A may play a role in M1/M2 transformation in the retina.

The signaling pathways through which Sema3A regulates M1/M2-like microglia transformation requires further study. Evidence has shown that the NF-κB signaling cascade regulates the production of pro-inflammatory mediators and contributes to the M1/M2-like microglia transition [52, 53]. Sema3A enhances LPS-induced acute kidney injury by increasing Rac1 (a key factor for activation of NF-κB) and p65 and augments LPS-induced macrophage activation and cytokine production in a plexin-A4–dependent manner [24, 54]. Previous studies confirmed that retinal TLR4 expression is increased in a mouse model of ONC [55]. TRIF knockout (KO) inactivates the NF-κB signaling pathway and reduces pro-inflammatory cytokine release by inhibiting activation of microglia in mice retina [6]. The mechanism of endogenous degeneration of RGCs remains unclear. We speculate that Sema3A/Nrp1 is involved in activating the TLR4/NF-κB signaling pathway, inducing the polarization of microglia toward the M1-like phenotype. However, the molecular pathways remain to be verified. In brief, we find that Sema3A directly affects neuron polarity and inhibits their regeneration, participates in M1/M2-like microglia dynamics regulation, and increases RGCs apoptosis. Sema3A has both direct and indirect effects on RGCs, presenting a potential therapeutic target for optic nerve injury treatment.

## Conclusions

Our results provide the first evidence that retinal microglia are an important source of Sema3A protein post-ONC. Sema3A is associated with an increase in pro-inflammatory M1-like microglia, a decrease in anti-inflammatory M2-like cells, and increased RGCs apoptosis. Inhibition of Sema3A ameliorates RGCs apoptosis and promotes RGCs regeneration. Therefore, Sema3A could be a new therapeutic target for RGCs protection after optic nerve injury.

## Supplementary information

**Additional file 1: Fig. S1.** The effect of anti-Sema3A in ameliorating RGCs apoptosis. (A) Representative confocal images showing immunofluorescent staining of TUNEL (green) and Iba1 (red) with Hoechst (blue) nuclear staining in retina tissue with or without anti-Sema3A treatment at and 7 days post-injury. Scale bar = 25 μm. (B) Western Blot and quantitative analysis show expression of BCL-2, BAX expression in retina tissue at 3 days and with or without anti-Sema3A treatment at 7 days post-injury. β-actin was used as a loading control. (Mean ± SEM, n = 5)

## Data Availability

All data in this study are included in this manuscript.
