# Supplementary figures and images for: Semaphorin3A increases M1-like microglia and retinal ganglion cell apoptosis after optic nerve injury
Source: Cell Biosci. 2021 May 26;11:97. doi: 10.1186/s13578-021-00603-7 (PMC8157735; doi:10.1186/s13578-021-00603-7)

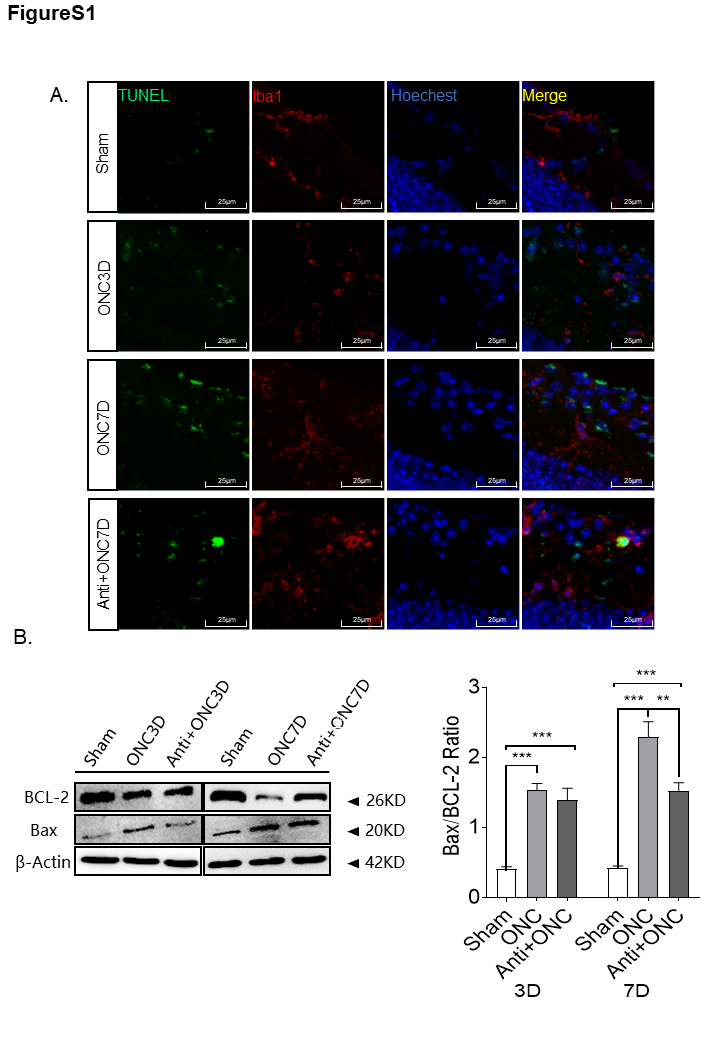

Supplement: Supplementary file 1 — Additional file 1: Fig. S1. The effect of anti-Sema3A in ameliorating RGCs apoptosis. (A) Representative confocal images showing immunofluorescent staining of TUNEL (green) and Iba1 (red) with Hoechst (blue) nuclear staining in retina tissue with or without anti-Sema3A treatment at and 7 days post-injury. Scale bar = 25 μm. (B) Western Blot and quantitative analysis show expression of BCL-2, BAX expression in retina tissue at 3 days and with or without anti-Sema3A treatment at 7 days post-injury. β-actin was used as a loading control. (Mean ± SEM, n = 5) [file 13578_2021_603_MOESM1_ESM.tif]
